# Limited Impact of 6-Mercaptopurine on Inflammation-Induced Chemokines Expression Profile in Primary Cultures of Enteric Nervous System

**DOI:** 10.1007/s11064-021-03324-y

**Published:** 2021-04-16

**Authors:** Jan Kneusels, Meike Kaehler, Ingolf Cascorbi, Thilo Wedel, Michel Neunlist, Ralph Lucius, François Cossais

**Affiliations:** 1 Institute of Anatomy, Kiel University, Kiel, Germany; 2 Institute of Experimental and Clinical Pharmacology, University Hospital Schleswig-Holstein, Kiel University, Kiel, Germany; 3 Inserm UMR 1235, University of Nantes, Nantes, France

**Keywords:** 6-Mercaptopurine, Enteric nervous system, Enteric glial cells, CXC motif ligand chemokines, Inflammatory bowel diseases

## Abstract

Increasing evidences indicate that the enteric nervous system (ENS) and enteric glial cells (EGC) play important regulatory roles in intestinal inflammation. Mercaptopurine (6-MP) is a cytostatic compound clinically used for the treatment of inflammatory bowel diseases (IBD), such as ulcerative colitis and Crohn’s disease. However, potential impacts of 6-MP on ENS response to inflammation have not been evaluated yet. In this study, we aimed to gain deeper insights into the profile of inflammatory mediators expressed by the ENS and on the potential anti-inflammatory impact of 6-MP in this context. Genome-wide expression analyses were performed on ENS primary cultures exposed to lipopolysaccharide (LPS) and 6-MP alone or in combination. Differential expression of main hits was validated by quantitative real-time PCR (qPCR) using a cell line for EGC. ENS cells expressed a broad spectrum of cytokines and chemokines of the C-X-C motif ligand (CXCL) family under inflammatory stress. Induction of *Cxcl5* and *Cxcl10* by inflammatory stimuli was confirmed in EGC. Inflammation-induced protein secretion of TNF-α and Cxcl5 was partly inhibited by 6-MP in ENS primary cultures but not in EGC. Further work is required to identify the cellular mechanisms involved in this regulation. These findings extend our knowledge of the anti-inflammatory properties of 6-MP related to the ENS and in particular of the EGC-response to inflammatory stimuli.

**Supplementary Information:**

The online version contains supplementary material available at 10.1007/s11064-021-03324-y.

## Introduction

Inflammatory bowel diseases (IBD), such as Crohn’s disease and ulcerative colitis (UC), are characterized by chronic relapsing intestinal inflammation. While the incidence and prevalence of IBD are continuously increasing worldwide [1, 2], their etiology and pathogenesis still remain largely unknown. In recent years, a large body of evidence has grown indicating that complex interactions between genetic, environmental and microbial factors and the intestinal neuro-immune system are involved in development and maintenance of IBD [3, 4].

Nowadays, it is widely acknowledged that the release of inflammatory mediators, including a broad variety of cytokines and chemokines, plays an essential role in IBD by stimulating the recruitment and activation of different types of immune cells. For example, the chemokine ENA-78/Cxcl5 binds the receptor Cxcr2 expressed by neutrophils that contribute to tissue damage in acute inflammation [5, 6]. In chronic inflammation, most tissues show an elevated expression of MIG/Cxcl9, IP-10/Cxcl10, and of their receptor Cxcr3 expressed by T-cells [5, 7]. Interestingly, Cxcl5, Cxcl9, Cxcl10, and their receptors have all been involved in the inflammatory processes underlying IBD [6–9]. However, despite significant advances, further research is needed to identify the cellular actors underlying intestinal inflammation and find potential modulators of chemokines-related pathways in IBD.

In recent years, the enteric nervous system (ENS) has been identified as an important regulator of inflammatory reactions in the intestinal tract [10]. Composed of enteric neurons and enteric glial cells (EGC), both cell types respond to inflammatory stimuli and participate in the inflammatory response by expressing pro- and anti-inflammatory factors [11]. For instance, enteric neurons are an important source of the pro-inflammatory cytokine TNF-α in the gut [12]. EGC are similarly involved in the regulation of inflammatory processes in the gastro-intestinal tract [13]. Indeed, EGC response to inflammatory insults is characterized by altered expression of enteric glial markers, e.g. S100β and GFAP, in acute and chronic intestinal inflammatory disorders [14–18]. Furthermore, EGC contribute to the regulation of intestinal inflammatory status via production of the polyunsaturated fatty acid metabolite 15-hydroxyeicosatetraenoic acid (15-HETE) in IBD patients [19] and release pro-inflammatory cytokines, including IL-6 [20] and IL-8 [21], under inflammatory conditions. Despite these important findings, little is known about the profile of inflammatory mediators expressed by the ENS or therapeutic agents potentially mitigating ENS response to inflammatory stress.

Currently, there is no cure for IBD and treatment relies on symptom management and immunomodulatory drugs such as 6-mercaptopurine (6-MP) [22, 23]. 6-MP is a cytostatic agent that is metabolized through complex mechanisms to 6-thioguanine nucleotides, which inhibits DNA synthesis by acting as a purine analogue [22, 24]. Recent evidences have been provided in support of direct anti-inflammatory functions of 6-MP, occurring independently of its cytostatic activity. Indeed, 6-MP has been shown to promote T-cell cycle arrest and apoptosis in the Jurkat human T lymphocyte cell line [25]. Furthermore, 6-MP regulates the transcriptional activities of the orphan nuclear receptors NR4A1-3, which are involved in the regulation of inflammatory reactions and neoplasia [26, 27]. Recently, 6-MP has been shown to inhibit the response of microglia to LPS-induced inflammation through PI3K/Akt/mTOR signaling-mediated translational regulation, suggesting potential anti-inflammatory effects in glial cells [28]. However, potential anti-inflammatory properties of 6-MP on the ENS have not been yet evaluated.

In the present study, we first aimed to characterize the transcriptional response of the ENS and EGC to inflammatory stimuli and second, to evaluate the potential anti-inflammatory effects of 6-MP on these cell types.

## Material and Methods

### Primary Cultures of ENS

Experiments were performed in the Sox10flox mouse line [29] in agreement with the local Ethics Committee (V 242-70056/2015(91-7/15)) and in accordance with the 3R principles (Replacement, Reduction and Refinement) to reduce the global number of animals sacrificed at our institute. Genetic modification of this mouse line was not relevant for the study. Murine primary cultures of ENS, composed of mixed cell population containing enteric neurons and EGC, were established based on previously published protocols for rat [12, 30] and mouse [31], with minor modifications. Briefly, removed gut segments of e12.5–e14.5 mouse embryos were dissected under a stereomicroscope using fine forceps. Tissue was mechanically minced using a chirurgical scalpel, placed in suspension in 2 ml DMEM/HAM F12 (1:1, Pan Biotech) medium and digested with 0.1% trypsin (Sigma-Aldrich) for 15 min at 37 °C. Cells were then treated with 0.01% DNase I (Sigma-Aldrich) for 15 min at 37 °C. Reaction was stopped by addition of DMEM/HAM F12 (1:1) medium supplemented with 10% v/v fetal calf serum (FCS, Pan-Biotech). Cells were seeded at a density of 4 × 10^5^ cells per well on 24-wells Cell + plates (Sarstedt). After 24 h, the medium was replaced by FCS-free DMEM/HAM F12 (1:1) supplemented with N2 (Pan Biotech). Primary cultures were composed of about 14–30% EGCs, and 4–10% enteric neurons (personal observation, determined by immunohistochemistry). Cells were allowed to grow for further 24 h before treatment. Cells were incubated with 10 or 50 µM 6-MP (Sigma-Aldrich) for 16 h before treatment with LPS (10 ng/ml, Sigma-Aldrich) for further 6 h.

### Enteric Glial Cell Line

The EGC line JUG2, which derives from rat ENS primary culture, was used for this study [32]. Cells were maintained in DMEM medium supplemented with 10% v/v FCS (Pan-Biotech). Cells were seeded at 3 × 10^5^ cells per well in 12-wells plates (Sarstedt). Cells were incubated with 10 or 50 µM 6-MP (Sigma-Aldrich) for 16 h before treatment with LPS (10 ng/ml, Sigma-Aldrich) or combination of TNF-α and IL1β (T + I, 100 ng/ml respectively, Immunotools) for further 24 h. Analyses were performed on at least five independent experiments.

### RNA Isolation and Real-Time Quantitative PCR

Total RNA extraction was performed using Nucleozol (Macherey–Nagel) and extracted RNA was stored at − 80 °C until further processing. Reverse transcription was performed on 1 µg of total RNA using the Revert Aid reverse transcription kit (Thermo-Fisher Scientific) according to manufacturer’s recommendations. Quantitative PCR (qPCR) was performed on 5 ng cDNA using the Evagreen Supermix (Solis biodyne) or the qPCR Master Mix Plus (Eurogentec) on an ABI Prism 7500 fast Real-Time PCR cycler (Life Technologies). The housekeeping genes RPS6 or HPRT were used for normalization. Primer sequences are listed in Supplementary Table 1. Relative quantitation to control conditions was performed using the ΔΔCt method and normalization to inflammatory conditions (LPS or T + I, respectively, set to 100%) was done.

### Microarray Analysis

Genome-wide expression analysis was performed on 100 ng total RNA obtained from three independent experiments using Clariom S arrays (Thermo-Fisher Scientific) according to manufacturer’s recommendations. Data were analyzed using the Transcriptome Analysis Console v4.0.1.36 and are available at the GEO database (GSE171308). Results were considered statistically significant for p-value < 0.05 and fold change ≥  ± 2.

### Comparison to Human Data-Set

Genome-wide expression data-sets GSE92415 [33] and GSE87466 were obtained from the Gene expression omnibus (GEO) database. Gene expression in colon mucosal tissue of healthy controls was compared to UC patients (GSE92415 control vs. UC patients placebo group, n = 21 and n = 52 respectively and GSE87466 control vs. UC patients, n = 21 and n = 27 respectively) using the GEO2R online tool. Venn Diagram and analysis of most significant overlapping differentially expressed genes (DEG, fold-change > 4 and FDR-p-value < 0.05) between data-sets was performed using the online tool Venn (http://bioinformatics.psb.ugent.be/webtools/Venn/).

### Gene Ontology and Pathways Analysis

Pathways and disease association analyses were performed using the online tools DAVID (Version 6.8) and Toppgene respectively. Gene network analysis was performed using Cytoscape (Version 3.8.2) running the plugin GeneMania [34].

### ELISA

Cytokine concentrations in cell culture supernatants were measured employing enzyme-linked immunosorbent assay (ELISA) kits for rat and mouse TNF-α (Thermo-Fisher Scientific), rat and mouse IL-6 (Thermo-Fisher Scientific), mouse CXCL1 (Peprotech), mouse CXCL2 (Peprotech), mouse CXCL5 (Sigma-Aldrich), mouse CXCL9 (Sigma-Aldrich), mouse CXCL10 (Peprotech), rat CXCL5 (Sigma-Aldrich), rat CXCL9 (Thermo-Fisher Scientific) and rat CXCL10 (Peprotech). Assays were performed according to manufacturer’s instructions. The absorbance was measured at 450 nm.

### Statistical Analyses

Statistical analyses were performed using the Prism software (Graphpad Version 8.4.2). Student *t*-test was used to perform comparison between two groups. Normality testing was performed using D’Agostino and Pearson test. In case of parametric distribution, ANOVA followed by Tukey’s posttest was performed to compare three groups or more and Friedman’s test followed by Dunn’s posttest was performed otherwise. Results were considered significant for p < 0.05.

## Results

### LPS Induces a Complex Profile of Inflammatory Mediators in ENS Primary Cultures

**Fig. 1:**
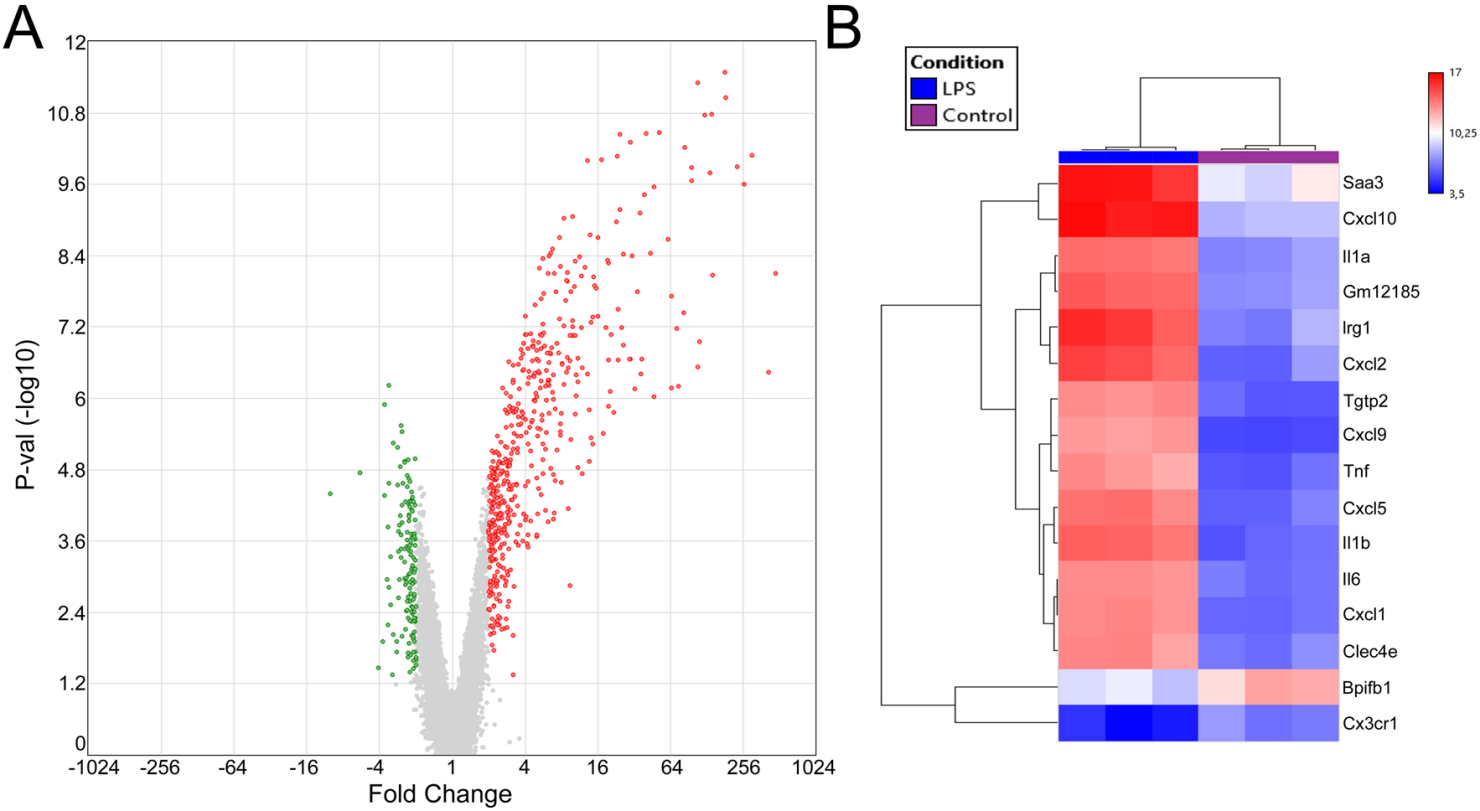
Impact of LPS treatment on the transcriptome of ENS primary cultures. ENS primary cultures were incubated with LPS at 10 ng/ml for 6 h, results for three independent experiments are shown. **a** Volcano plots showing the differential gene expression between control and LPS conditions. Red and green dots represent significant up- and down-regulated genes respectively (fold change >  ± 2 and FDR-p-value < 0.05). **b** Hierarchical clustering of the main differentially expressed genes (fold change > 100 or <  − 2) between control and LPS-treated ENS primary cultures

In order to gain better understanding of ENS involvement in intestinal inflammation, we performed microarray-based gene expression profiling comparing LPS-treated ENS primary cultures to untreated controls. Differential transcriptional regulation was observed for 628 genes, including 456 up-regulated and 90 down-regulated genes (Fig. 1a and Supplementary Table 2).

**Fig. 2:**
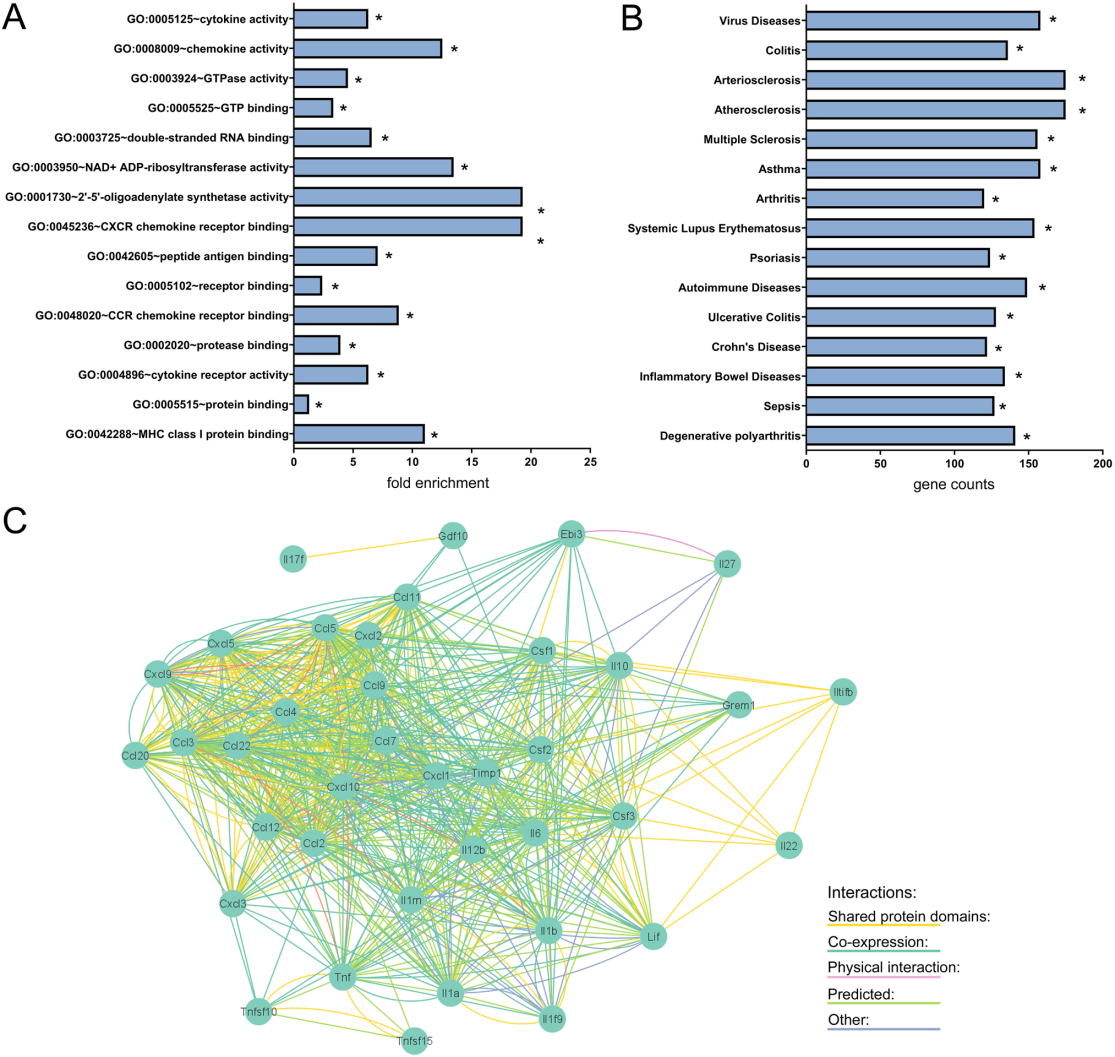
Gene ontology (GO) analyses of LPS-treatment on ENS primary cultures. Primary cultures of ENS were incubated with LPS at 10 ng/ml for 6 h. **a** Top 15 GO-molecular pathways fold enrichment in LPS-treated ENS primary cultures in comparison to control. **b** Gene counts present in the top15 most significant diseases associated with the altered transcriptomic profile observed in LPS-treated ENS primary cultures. **c** Interaction network of cytokines and chemokines hits (GO:0005125) significantly induced by LPS in ENS primary cultures.*FDR-p-value < 0.05

**Fig. 3:**
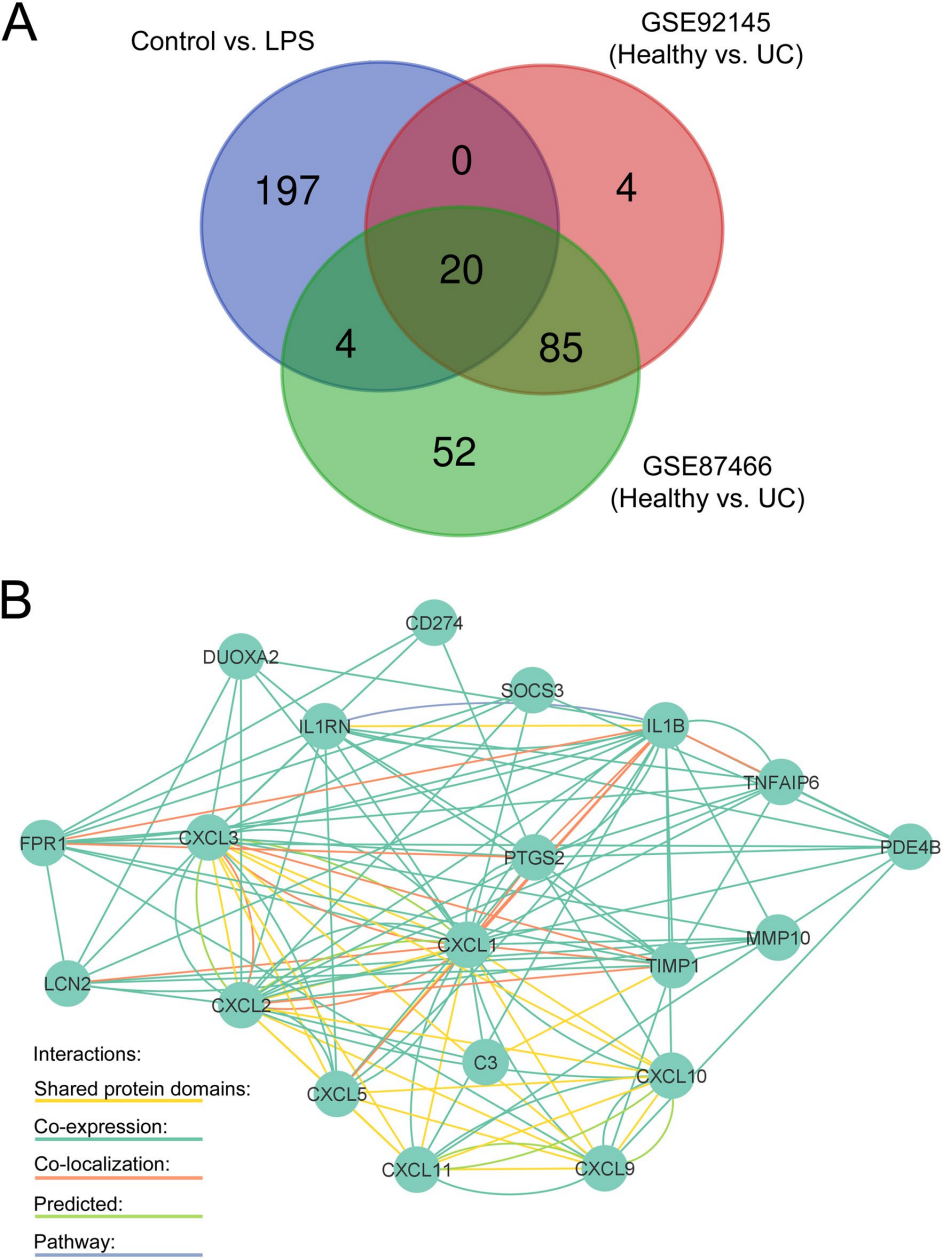
Overlap between LPS-induced ENS primary cultures and ulcerative colitis (UC) transcriptomic profiles. **a** Venn diagram showing overlapping differentially expressed genes (DEGs) observed in LPS-treated ENS primary cultures (control vs. LPS) in comparison to DEGs observed in mucosal tissues of UC patients (GSE92415, GSE87466, control vs. UC, for fold-change > 4 and FDR-p-value < 0.05). **b** Interaction network of overlapping DEGs observed in **a**

More particularly, expression of the pro-inflammatory cytokines *TNF-α*, *IL-6* and *IL-1β* was clearly induced after LPS stimulation (Fig. 1a, b and Supplementary Table 2). Interestingly, a large set of chemokines of the CXC-motif family were also significantly up-regulated after LPS treatment for 6 h, with the chemokines *Cxcl1*, *Cxcl2*, *Cxcl5*, *Cxcl9* and *Cxcl10* showing more than 100 fold upregulation (Fig. 1b and Supplementary Table 2). Gene ontology analyses confirmed the induction of main inflammatory pathways, as well as the activation of cytokine and chemokine pathways (Fig. 2a–c, Supplementary Table 3). In particular, cytokine activity appeared as the most significantly affected molecular pathway with a set of 38 cytokines showing altered expression (GO:0005125, Fig. 2c, Supplementary Table 4). Chemokine activity was found to be the second most significant module related to gene ontology molecular functions, with a set of 17 chemokines being enriched in ENS primary cultures after stimulation with LPS (GO:0008009, Supplementary Table 4). The LPS-induced differentially expressed genes were significantly associated with transcriptomic profiles observed in intestinal inflammatory disorders such as colitis (C0009319) and IBD (C0021390, Fig. 2b, Supplementary Table 5). We then compared the differential transcriptomic profile induced by LPS in ENS primary cultures to the transcriptomic profiles observed in intestinal tissue of UC patients (GSE92415 and GSE87466, control vs. UC). Overlap was observed for a total of 20 genes when comparing most significantly DEG between the different data-sets (Fig. 3a). In particular, genes related to chemokine activity, including Cxcl1, Cxcl2, Cxcl5, Cxcl9 and Cxcl10 were significantly enriched amongst the overlapping hits (Fig. 3b, Supplementary Table 6).

### 6-MP has Limited Impact on LPS-Induced Transcriptomic Profile in ENS Primary Cultures

**Fig. 4:**
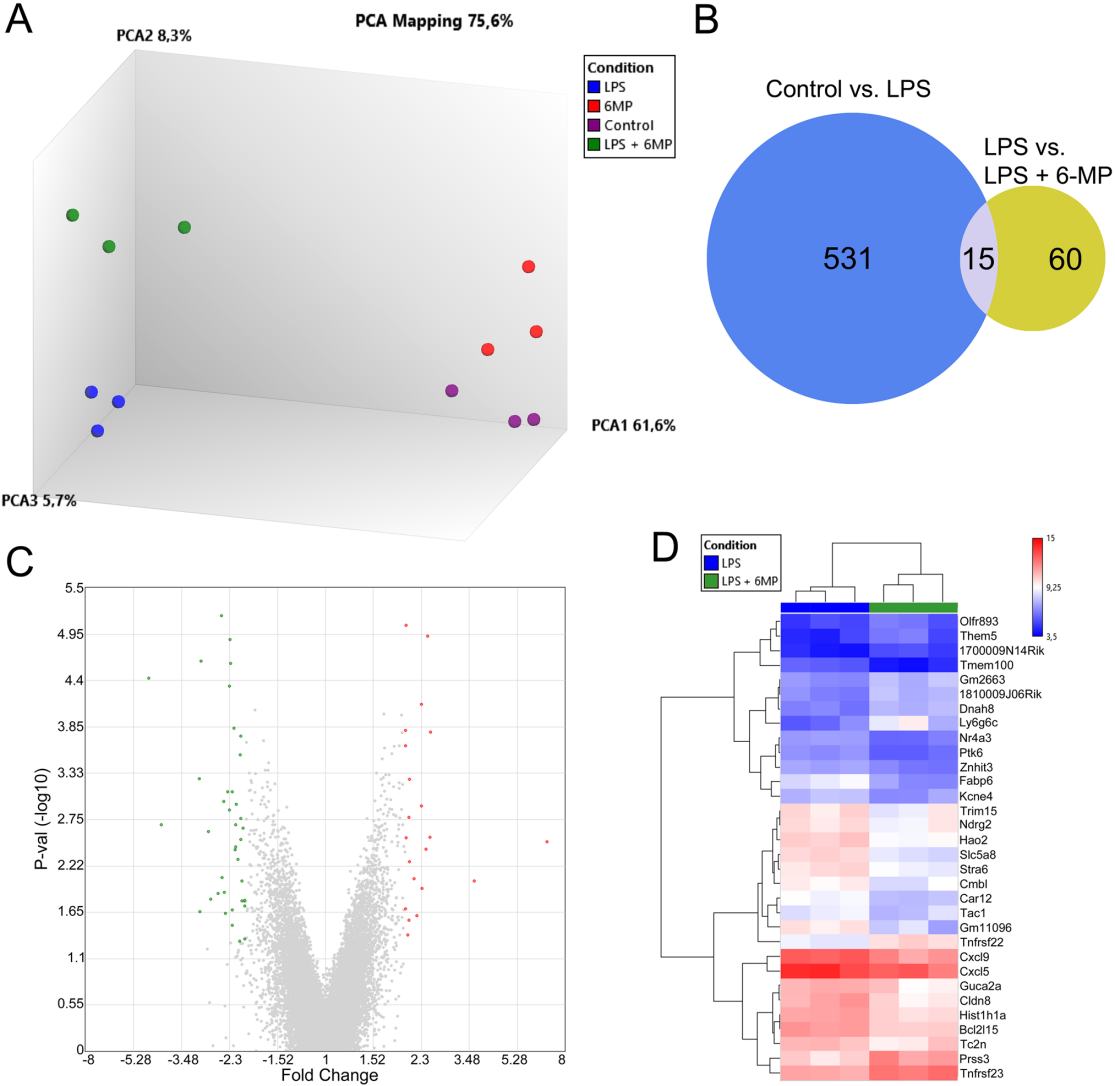
Impact of 6-MP on LPS-induced transcriptomic alterations in ENS primary cultures. Primary cultures of ENS were incubated with LPS at 10 ng/ml for 6 h after pre-treatment for 16 h with 6-MP at 50 µM and compared to untreated control conditions. Principal component analysis (**a**) showing the differential gene expression between primary ENS cultures in control condition or treated with LPS and 6-MP alone or in combination in three independent experiments. Axis 1, 2 and 3 represent 61.6, 8.3 and 5.7% of the total observed variance respectively. Venn diagram (**b**) representing the number of genes differentially expressed and overlapping in LPS vs. control (blue) and in LPS vs. LPS + 6-MP (yellow) conditions. Volcano plots showing the differential gene expression between LPS and LPS + 6-MP conditions (**c**). Red and green dots represent up- and down-regulated genes respectively (fold change >  ± 2 and p-value < 0.05). **d** Hierarchical clustering of the main differentially regulated genes (fold change > 2.2 and <  − 2.2) for ENS primary cultures co-treated with LPS and 6-MP

As observed in principal component analysis, 6-MP treatment had low impact per se on the transcriptomic profile of ENS primary cultures in comparison to control conditions (Fig. 4a, compare 6-MP to control group, Supplementary Table 7). Under LPS-stimulation, based on their transcriptomic profile, cells pre-treated with 6-MP formed an individual cluster, which was clearly distinct from control or LPS conditions (Fig. 4a). This distinct transcriptomic profile relied on co-altered expression of 60 genes between LPS-treated ENS primary cultures and cultures treated with LPS and 6-MP in combination, although single differential expression of these genes did not remain significant after FDR-correction (Fig. 4c, d, Supplementary Table 8). As indicated in the Venn diagram, amongst these 60 genes, only 15, including the chemokines *Cxcl5* and *Cxcl9*, also belonged to the group of genes upregulated by LPS, (Fig. 4b, Supplementary Table 9).

### 6-MP has Limited Impact on LPS-Induced Expression of Chemokines in ENS Primary Cultures

**Fig. 5:**
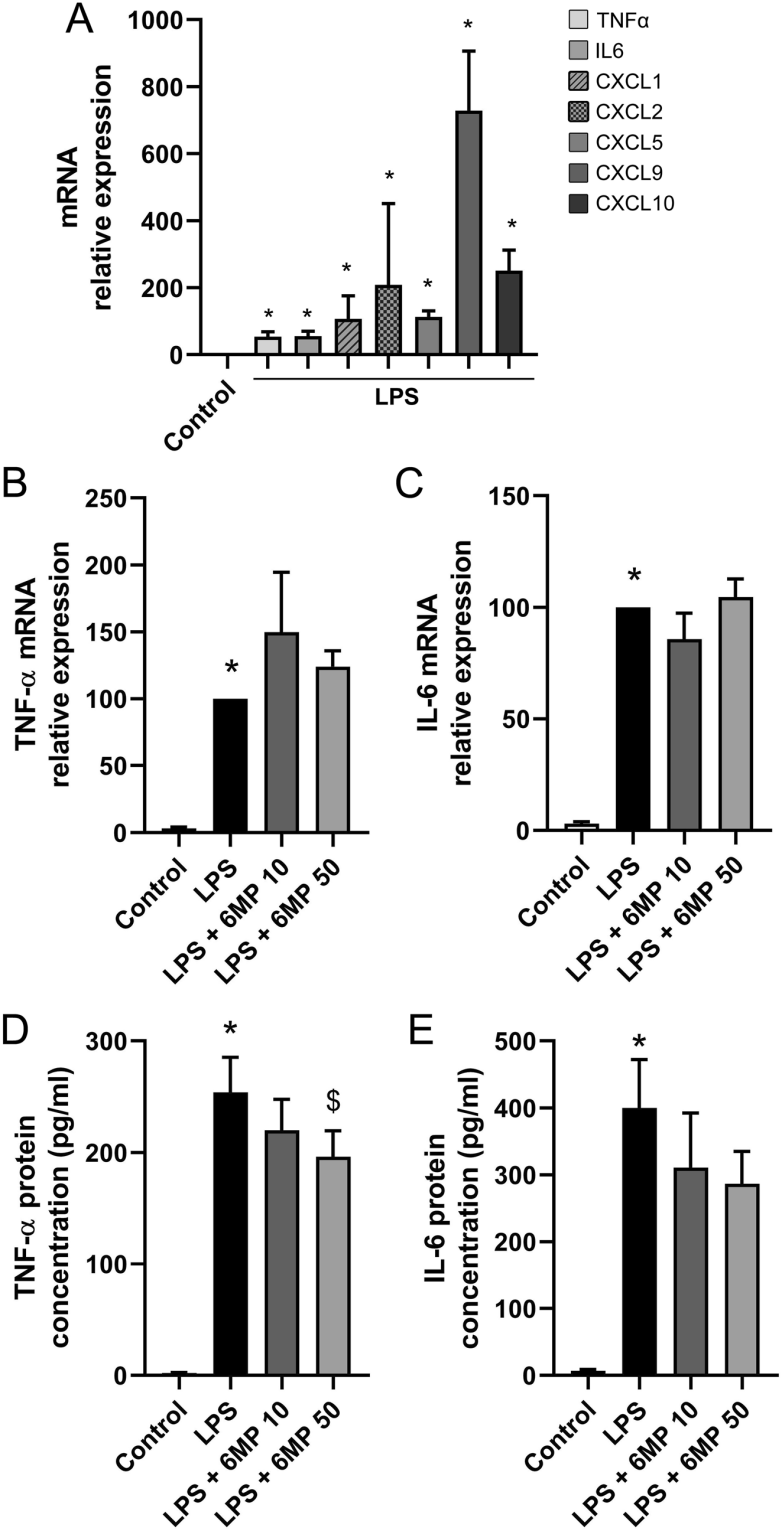
Impact of 6-MP on LPS-induced expression of selected cytokines and chemokines in ENS primary cultures. LPS at 10 ng/ml for 6 h induced the mRNA expression of the inflammatory mediators *TNF-α*, *IL-6*, *Cxcl1*, *2*, *5*, *9* and *10*, as determined by qPCR (**a**, n = 9–11, data were normalized to control expression). Impact of pre-incubation with 6-MP at 10 and 50 µM for 16 h on LPS-induced mRNA expression of *TNF-α* (**b**) and *IL-6* (**c**) was determined by qPCR (n = 7–11). Data were normalized to LPS conditions. ELISA was used to measure the impact of 6-MP at 10 and 50 µM on LPS-induced protein production of TNF-α (**d**) and IL-6 (**e**, n = 11). Data were normalized to LPS conditions. *p < 0.05 in comparison to control. ^$^p < 0.05 in comparison to LPS

**Fig. 6:**
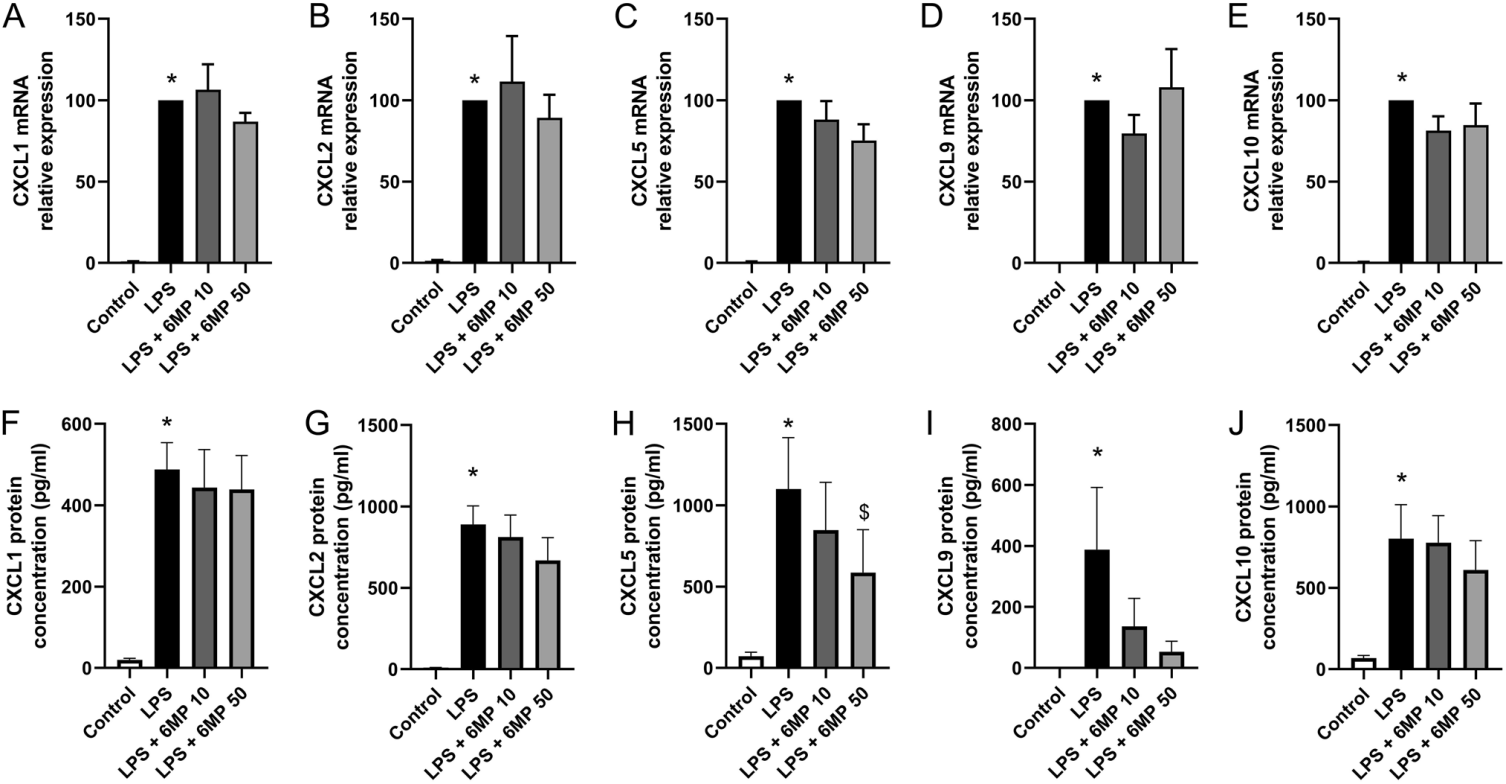
Impact of 6-MP on LPS-induced expression of chemokines *Cxcl1*, *2*, *5*, *9* and *10* in ENS primary cultures. Impact of pre-incubation with 6-MP at 10 and 50 µM for 16 h on LPS-induced (10 ng/ml, 6 h) mRNA expression of *Cxcl1* (**a**), *Cxcl2* (**b**), *Cxcl5* (**c**), Cxcl9 (**d**) and *Cxcl10* (**e**) was determined by qPCR (n = 9–11). Data were normalized to LPS conditions. Protein production of Cxcl1 (**f**), Cxcl2 (**g**), Cxcl5 (**h**), Cxcl9 (**i**) and Cxcl10 (**j**) was determined by ELISA (n = 10). *p < 0.05 in comparison to control. ^$^p < 0.05 in comparison to LPS

Quantitative PCR was used to validate the results of the genome-wide expression analysis, with a particular focus on TNF-α, IL-6 and the chemokines *Cxcl1*, *Cxcl2*, *Cxcl5*, *Cxcl9* and *Cxcl10*. LPS stimulation resulted in upregulation of *TNF-α* and *IL-6* expression by 53.8 ± 15.2 and 55.0 ± 15.4-fold respectively after 6 h (Fig. 5a). Similarly, expression of the chemokines *Cxcl1* and *Cxcl2* increased by 107.1 ± 20.8 and 208.0 ± 81.0-fold respectively. *Cxcl5*, *Cxcl9* and *Cxcl10* mRNA expression in primary culture of ENS were upregulated by 112.6 ± 18.6, 728.8 ± 177.7, and 251.2 ± 61.5-fold respectively, confirming the results of the microarray (Fig. 5a). Pre-treatment with 10 or 50 µM 6-MP did not affect the LPS-induced mRNA expression of *TNF-α* and *IL-6* (Fig. 5b, c). LPS-induced TNF-α protein release was significantly reduced after pre-treatment with 50 µM 6-MP (Fig. 5d). LPS-induced IL-6 protein production was not significantly inhibited by pre-treatment with 50 µM 6-MP, as determined by ELISA (Fig. 5e). Pre-treatment of ENS primary cultures with 6-MP showed hardly any effects on the LPS-induced mRNA expression of *Cxcl1*, *Cxcl2*, *Cxcl5*, *Cxcl9* and *Cxcl10* (Fig. 6a–e). Pre-treatment with 50 µM 6-MP inhibited the LPS-induced protein production of Cxcl5 by 22.9% (Fig. 6h) but had no impact on the release of the chemokines Cxcl1, Cxcl2, Cxcl9 and Cxcl10 (Fig. 6f–j).

### Chemokines Expression is Upregulated by TNF-α and IL-1β in EGC

**Fig. 7:**
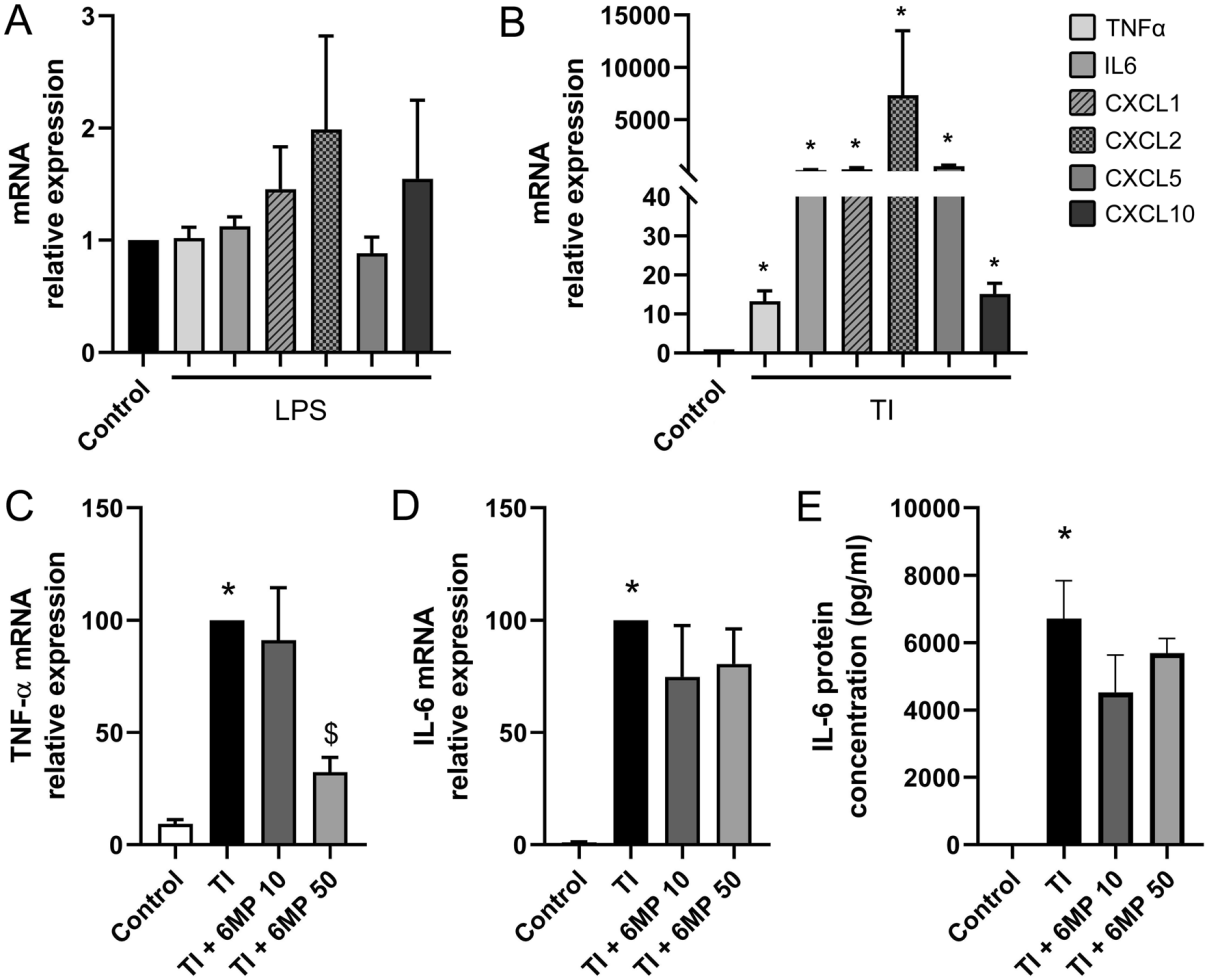
Impact of 6-MP on TI-induced expression of selected cytokines and chemokines in JUG2 cells. Impact of 10 ng/ml LPS (**a**) and combination of TNF-α and IL-1β (TI), 100 ng/ml respectively, for 24 h on mRNA expression of *TNF-α*, *IL-6*, *Cxcl1*, *Cxcl2*, *Cxcl5* and *Cxcl10*, as determined by qPCR (**b**, n = 4–6, data are normalized to untreated control expression). Impact of pre-incubation with 6-MP at 10 and 50 µM for 16 h on TI-induced mRNA expression of *TNF-α* (**c**) and *IL-6* (**d**) was determined by qPCR (n = 6). Data were normalized to TI conditions. ELISA was used to measure the impact of 6-MP at 10 and 50 µM on TI-induced protein production of IL-6 (**e**, n = 6, data are normalized to TI conditions). *p < 0.05 in comparison to control. ^$^p < 0.05 in comparison to TI

To address whether EGC may represent a potential source of inflammatory mediators, we analyzed the expression of TNF-α, IL-6, and the chemokines Cxcl1, Cxcl2, Cxcl5 and Cxcl10 in an EGC line (referred thereafter as JUG2) in response to inflammatory stimuli. LPS treatment failed to induce the expression of any of the cytokines or chemokines analyzed (Fig. 7a). Since the JUG2 cell line was not responsive to LPS, JUG2 cells were treated with TNF-α and IL-1β in combination (T + I), in presence or absence of 6-MP. At mRNA level, expression of *IL-6* was induced by 160.0 ± 49.6-fold by treatment with T + I for 24 h, whereas expression of *TNF-α* was induced by 13.2 ± 2.7-fold. The expression of the chemokines *Cxcl1*, *Cxcl2*, *Cxcl5* and *Cxcl10* was induced by 238.3 ± 66.1, 7333.0 ± 2521.0, 536.8 ± 104.9 and 15.1 ± 2.7-fold respectively by T + I after 24 h treatment in JUG2 cells (Fig. 7b). Pre-treatment of JUG2 cells with 50 µM 6-MP inhibited the T + I-induced mRNA expression of *TNF-α* by 67.7 ± 6.6% (Fig. 7c). Pre-treatment with 6-MP did not affect the T + I-induced IL-6 expression neither at mRNA nor at protein level (Fig. 7d, e).

### 6-MP has Limited Impact on the Expression of Chemokines Induced by TNF-α and IL-1β in EGC

**Fig. 8:**
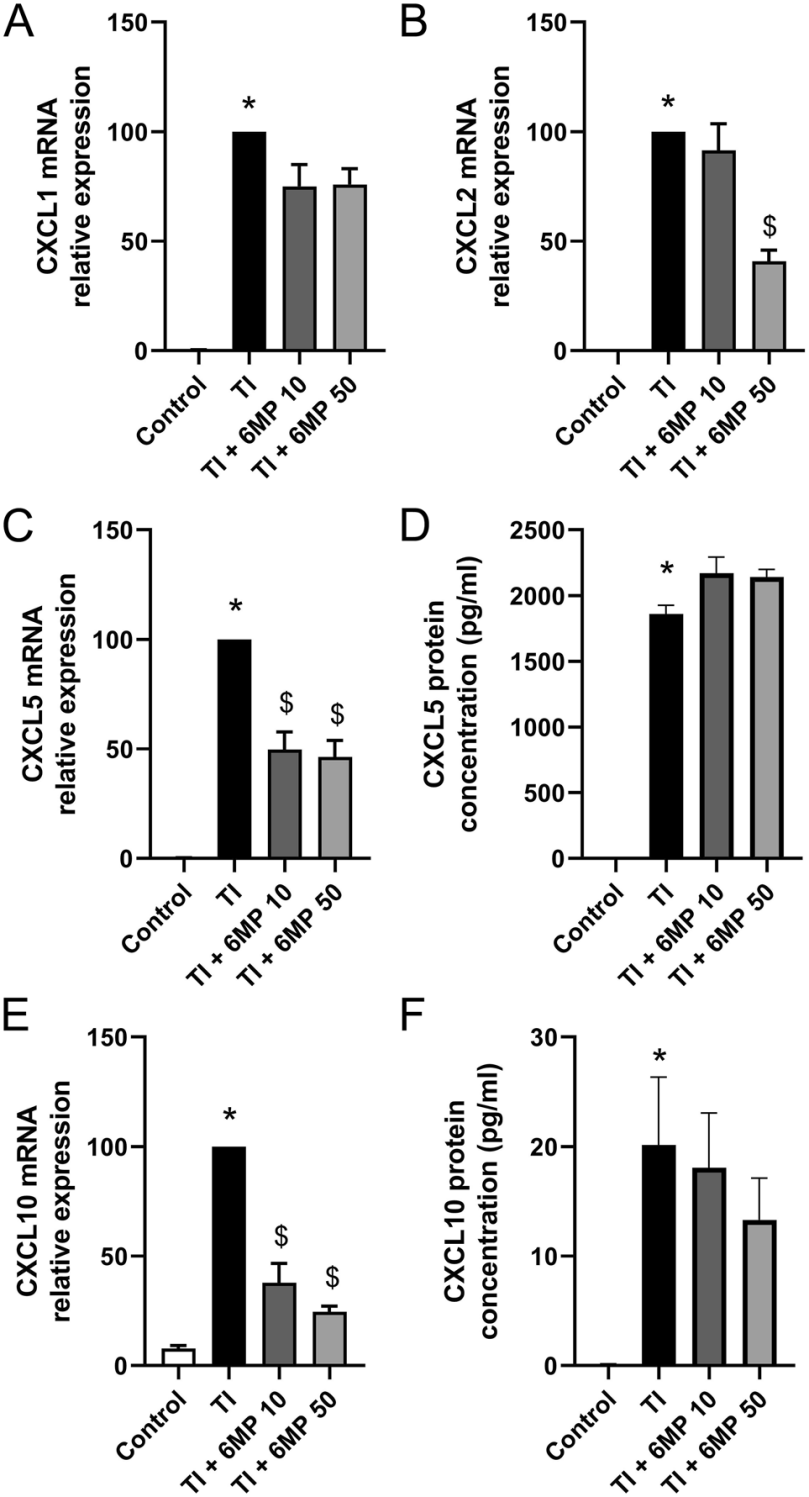
Impact of 6-MP on TI-induced expression of the chemokines *Cxcl1*, *Cxcl2*, *Cxcl5* and *Cxcl10* in JUG2 cells. Impact of pre-incubation with 6-MP at 10 and 50 µM for 16 h on JUG2 cells treated with TNF-α and IL-1β in combination (TI), 100 ng/ml respectively, for 24 h. Expression of *Cxcl1* (**a**), *Cxcl2* (**b**), *Cxcl5* (**c**) and *Cxcl10* (**e**) was determined by qPCR (n = 5–6). Data were normalized to TI conditions. Protein production of Cxcl5 (**d**) and Cxcl10 (**f**) was determined by ELISA (n = 6). *p < 0.05 in comparison to control. ^$^p < 0.05 in comparison to LPS

T + I-induced mRNA expression of *Cxcl1* and *Cxcl2* was reduced respectively by 24.2 ± 7.3 and 59.2 ± 5.1% by treatment with 50 µM 6-MP (Fig. 8a and b). T + I-induced mRNA expression of *Cxcl5* was inhibited by pre-treatment with 10 µM and 50 µM 6-MP by 50.3 ± 8.1 and 53.8 ± 7.6% respectively (Fig. 8c). However, no impact of 6-MP pre-treatment at either concentration was observed on Cxcl5 protein production (Fig. 8d). Similarly, pre-treatment with 10 µM and 50 µM 6-MP inhibited the T + I-induced mRNA expression of *Cxcl10* by 62.2 ± 8.9% and 75.4 ± 2.6% respectively but did not altered T + I-induced Cxcl10 protein release (Fig. 8e, f). Of note, no induction of Cxcl9 protein was detected after combined T + I treatment in JUG2 cells (data not shown).

## Discussion

Increasing evidences indicate that the ENS is an important regulator of intestinal inflammation. Over the last decades, several independent research groups have demonstrated that both enteric neurons and EGC constitute a source of inflammatory cytokines and chemokines [20, 21, 35]. In line with these results, we demonstrate that the ENS expresses a complex profile of inflammatory mediators in response to inflammatory stimuli. In particular, inflammatory stress in ENS primary cultures, as well as in EGC, induces the expression of a broad profile of chemokines, including Cxcl1, Cxcl2, Cxcl5, Cxcl9 and Cxcl10, extending the known inflammatory portfolio of these cell populations.

Enteric neuro-immune interactions are key mechanisms in IBD and induction of chemokine pathways by inflammatory stimuli within the ENS may play an important role in recruiting immune cell populations, such as T-lymphocytes [36], mast cells [37], or macrophages [38] in the intestinal wall. Rat ENS primary cultures have revealed as a suitable model to study ENS functions under inflammatory stress [12]. In our study, using mouse ENS primary cultures, ontology association analyses indicated that the observed LPS-induced inflammatory expression profile was significantly related to the transcriptomic profiles observed in colitis or IBD. This assumption was confirmed by comparing our results to intestinal transcriptomic profiles of UC patients, supporting the rational to use ENS primary cultures to study chemokines activation under inflammatory stress. Most particularly, these results support the involvement of broad chemokine pathways in the inflammatory active phases of IBD and are in line with recent data of the literature based on the analysis of additional publicly available genome-wide data-sets of UC patients [39]. In support of this data, expression of Cxcl1 [9] and Cxcl2 [40, 41] has also been shown to be increased in intestinal mucosal biopsies of IBD patients in additional independent studies. Similarly, Cxcl9 expression is up-regulated in UC [9], and Cxcl5 is one of the main chemokines expressed in intestinal tissue of Crohn’s disease patients [42]. Moreover, increased expression of Cxcl10 and its receptor Cxcr3 has been confirmed in intestinal biopsies of IBD patients [43].

Importantly, using the EGC cell line JUG2, we demonstrated that EGC themselves represent an important source of chemokines under inflammatory stress. In particular, although the JUG2 cell line remained insensitive to LPS stimulation, our data clearly indicates that Cxcl1, Cxcl2, Cxcl5 and Cxcl10 belonged to the main chemokines induced by inflammatory stress in EGC, although induced Cxcl10 production remained at relatively low levels in JUG2 cells. In line with our results, induced *Cxcl2* and *Cxcl10* expression has been previously demonstrated in primary cultures of human EGC after combined stimulation with LPS and interferon-γ [44]. Induced expression of Cxcl5 has also been observed in EGC-derived gliospheres after stimulation with LPS [35]. Interestingly, inflammation-induced expression of Cxcl5 has been observed in intestinal epithelial cells in IBD patients, as well as in the caco-2 cell line, but contribution of the ENS as a potential source of inflammatory mediators was not addressed in these early studies [45–47]. Although it remains unclear to which extend the ENS contributes to chemokines release in IBD patients, our results clearly indicate that ENS cell populations and in particular EGC may constitute a significant source of chemokines of the CXCL-family in intestinal tissues under inflammation.

6-MP has been widely used for the clinical treatment of IBD [23] and recent studies have given evidences for direct anti-inflammatory properties of 6-MP [25, 28, 48]. In particular, 6-MP was shown to inhibit the induced expression of *TNF-α*, *IL-6* and *Cxcl1* in airway epithelial cell lines under inflammatory stress [48]. Similarly, LPS-induced mRNA and protein expression of *TNF-α* is inhibited by 6-MP in microglia in a process involving the NF-κB pathway [28].

In our study, potential direct anti-inflammatory properties of 6-MP were rather limited both in EGC, as well as in ENS primary cultures. Indeed, in ENS primary cultures, protein expression of TNF-α and Cxcl5 were in part inhibited by pre-treatment with 6-MP, without any alterations of mRNA expression. These results suggest that 6-MP may lead to substantial post-transcriptional alterations of TNF-α and Cxcl5 release, without interfering directly with NF-κB transcriptional activity in this complex cellular model. On the opposite, 6-MP inhibition of inflammation-induced mRNA expression of *Cxcl5* and *Cxcl10* was not associated with any reduction of chemokines release in the JUG2 cell line. Although intriguing, the observed differences between primary cultures and JUG2 cells may rely on the differential activity of 6-MP in these models. For instance, anti-inflammatory effects of 6-MP observed in ENS primary cultures might rely on post-translational mechanisms involving gasdermin C (GSDMC), as gasdermins have been shown to regulate cytokines release at the plasma membrane [49]. Additionally, the mixed cell composition of ENS primary cultures may mitigate the effects of 6-MP by yet unidentified mechanisms. Characterization of the enzymatic machinery expressed by ENS cell population should bring important information on this aspect in future studies.

Until now, little is known about the molecular pathways involved in the mediation of direct anti-inflammatory effects of 6-MP. In microglia, decreased TNF-α production by 6-MP has been proposed to rely in part on orphan nuclear receptor Nur77-mediated transcriptional inhibition and concomitant translational repression involving the PI3K/Akt/mTOR pathways [28]. Noteworthy, neither increased expression of Nur77 nor activation of mTOR signaling pathways were observed in our transcriptomic analysis, suggesting that alternative cellular mechanisms are involved in the mediation of 6-MP effects in the ENS. However, further experiments are required to fully evaluate the potential involvement of these pathways in our model.

Taken together, our results suggest that the direct anti-inflammatory effects of 6-MP observed on the ENS in vitro may rather play a limited therapeutic role. Despite these limitations, our study supports the involvement of the ENS, and more particularly of EGC, as an important source of inflammatory mediators in the gut. Targeting of chemokine pathways has offered promising results for the treatment of IBD in pre-clinical studies [50–52] and further work is required to fully evaluate the contribution of ENS-derived inflammatory mediators in chronic and acute intestinal inflammatory disorders. This characterization may help to develop novel therapeutic strategies by targeting the ENS-mediated inflammatory response in IBD and other intestinal inflammatory disorders.

## Supplementary Information

Below is the link to the electronic supplementary material.

Supplementary file1 (XLSX 150 kb)
